# Characterization and functional analysis of *GhWRKY42*, a group IId WRKY gene, in upland cotton (*Gossypium hirsutum* L.)

**DOI:** 10.1186/s12863-018-0653-4

**Published:** 2018-07-30

**Authors:** Lijiao Gu, Hengling Wei, Hantao Wang, Junji Su, Shuxun Yu

**Affiliations:** State Key Laboratory of Cotton Biology, Institute of Cotton Research, Chinese Academy of Agricultural Sciences, Anyang, Henan People’s Republic of China

**Keywords:** *GhWRKY42*, *Arabidopsis*, Leaf senescence, VIGS, Cotton, Plant height

## Abstract

**Background:**

WRKY transcription factors (TFs) participate in various physiological processes of plants. Although WRKY genes have been well studied in model plants, knowledge of the functional roles of these genes is still extremely limited in cotton.

**Results:**

In this study, a group IId WRKY gene from cotton, *GhWRKY42*, was isolated and characterized. Our data showed that *GhWRKY42* localized to the nucleus. A transactivation assay in yeast demonstrated that *GhWRKY42* was not a transcriptional activator. A β-glucuronidase (GUS) activity assay revealed that the promoter of *GhWRKY42* showed fragment deletion activity in *Nicotiana tabacum* and was mainly expressed in the roots, stems and leaves of *ProGhWRKY42::GUS* transgenic *Arabidopsis* plants. Quantitative real-time PCR (qRT-PCR) analysis indicated that *GhWRKY42* was up-regulated during leaf senescence and was induced after exposure to abiotic stresses. Constitutive expression of *GhWRKY42* in *Arabidopsis* led to a premature aging phenotype, which was correlated with an increased number of senescent leaves, reduced chlorophyll content and elevated expression of senescence-associated genes (SAGs). In addition, virus-induced gene silencing (VIGS) was used to silence the endogenous *GhWRKY42* gene in cotton, and this silencing reduced plant height.

**Conclusions:**

Our findings indicate that *GhWRKY42* is involved in abiotic stress responses, premature leaf senescence and stem development. This work establishes a solid foundation for further functional analysis of the *GhWRKY42* gene in cotton.

**Electronic supplementary material:**

The online version of this article (10.1186/s12863-018-0653-4) contains supplementary material, which is available to authorized users.

## Background

Plants are constantly challenged by various factors that affect plant growth and development throughout their life cycle. To combat these challenges, some responsive genes, including WRKY transcription factors (TFs), are induced to help plants adapt through physiological and morphological changes [1]. WRKY TFs are plant-specific proteins and constitute one of the largest TF families in plants [2]. WRKY TFs share the common feature of a highly conserved WRKY domain that consists of the peptide sequence motif WRKYGQK at the N-terminus and a zinc-finger-like motif at the C-terminus [3]. WRKY TFs have one or two conserved WRKY domains, and these domains contain a Cx4-5Cx22-23HxH or Cx7Cx23HxC zinc-finger-like motif. Based on the number of conserved WRKY domains and the structural characteristics of the zinc-finger-like motifs, WRKY TFs can be categorized into group I, group II or group III. Group II can be further divided into subgroups IIa, IIb, IIc, IId and IIe [3–7]. WRKY TFs can recognize and bind to the W-box sequences [TTGAC(C/T)] in the promoter region of target genes to participate in regulatory networks [8].

In plants, WRKY TFs are mainly involved in defense responses, trichome development, plant growth and development and leaf senescence [9]. Various TFs are involved in modulating leaf senescence, and 1533 TFs have been identified via leaf senescence transcriptome analyses in *Arabidopsis* [10, 11]. WRKY TFs are quantitatively important members of those TFs involved in leaf senescence [11]. In *Arabidopsis*, *AtWRKY6* is associated with the senescence process by targeting the promoter of the *SIRK* gene, which likely encodes a signaling component related to leaf senescence [12]. *AtWRKY45* was recently reported to interact with the DELLA protein *RGA-LIKE1* (*RGL1*) and to directly target the *SAG12*, *SAG13*, *SAG113* and *SEN4* genes, to positively modulate leaf senescence via the gibberellic acid-mediated signaling network [13]. In rice, *OsWRKY42* promotes senescence in transgenic rice plants by binding to the promoter of *OsMT1d* to repress ROS scavenging [14]. *OsWRKY23* is markedly increased during dark-induced leaf senescence, and *OsWRKY23*-overexpressing lines can accelerate leaf senescence under dark conditions [15]. Furthermore, *TaWRKY7* from wheat can significantly promote senescence in transgenic *Arabidopsis* under dark conditions [16].

According to previous reports, WRKY TFs are thought to be involved in the regulation of plant tissue growth and development. For example, *VvWRKY2* is specifically expressed in the lignified cells of young grapevine stems, and overexpression of *VvWRKY2* in *N. tabacum* affects the lignin biosynthesis pathway, thus influencing xylem development [17]. Li et al. reported that *Atwrky13* mutants exhibit weaker stems due to altered development of parenchyma cells [18]. Another WRKY TF, *WRKY71*/*EXB1*, positively regulates plant branching by controlling axillary meristem initiation and bud activities [19]. In addition, the pollen-specific WRKY TF *AtWRKY34* is phosphorylated by two mitogen-activated protein kinases, MPK3 and MPK6, in the regulation of male gametogenesis [20]. Furthermore, emerging evidence has demonstrated that WRKY TFs are widely involved in stress responses. For example, *GhWRKY40* is involved in pathogen responses [21], and *GhWRKY68* is involved in salt and drought stress responses [22]. These reports further emphasize the importance of studying WRKY TFs.

Cotton (*Gossypium hirsutum*) is an important economic crop that is widely cultivated around the world. As a significant source of fiber, oil and biofuel products, cotton has become an important industrial raw material. In field production, the growth and yield of cotton are severely restricted by both external environmental factors and internal factors. A growing number of studies have shown that WRKY TFs play important roles in the responses to these factors. Therefore, it is particularly important to study the functional roles of WRKY genes in cotton. In the present study, a group IId WRKY gene, *GhWRKY42*, was isolated and characterized. We performed a preliminary analysis of the gene structure, evolutionary relationships and expression patterns of *GhWRKY42*. Overexpression of *GhWRKY42* in *Arabidopsis* accelerated leaf senescence. In addition, silencing *GhWRKY42* in VIGS plants significantly reduced plant height.

## Results

### Sequence and evolutionary analysis of *GhWRKY42*

**Fig. 1:**
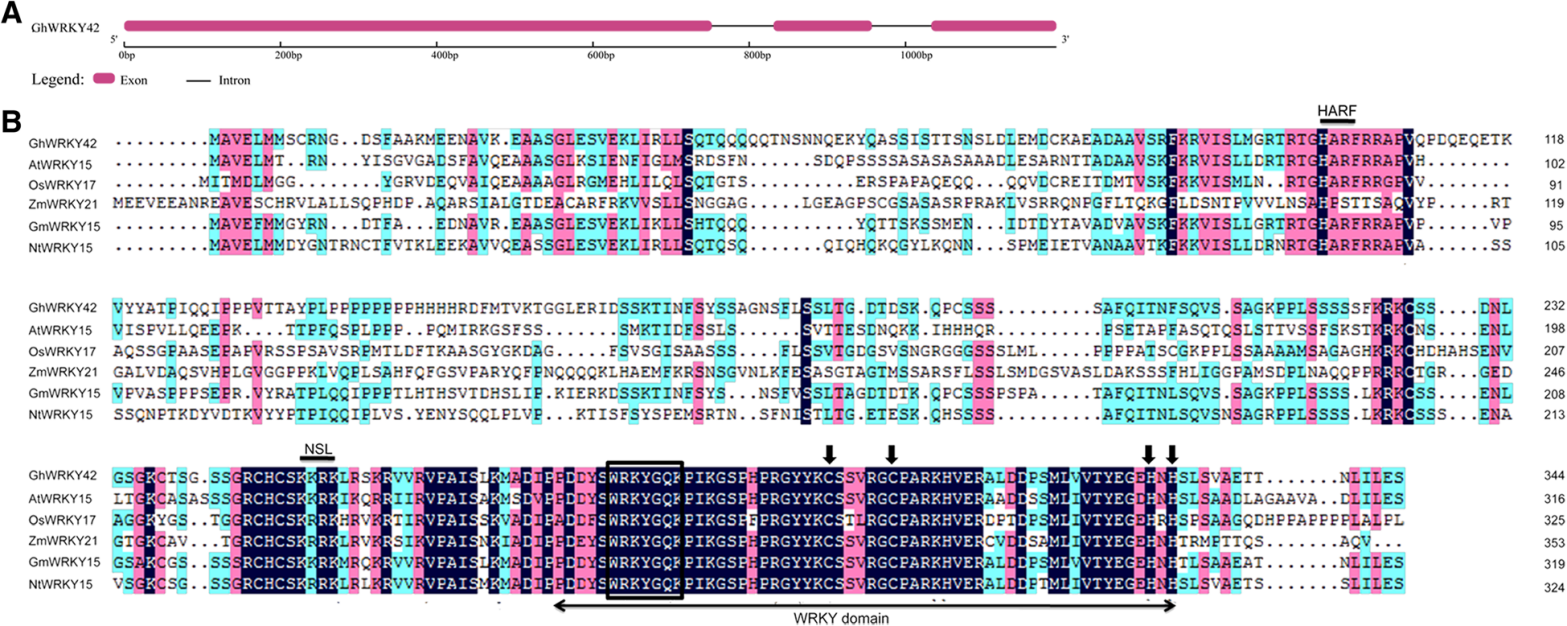
Gene structure and sequence analysis of *GhWRKY42*. **a** Gene structure of *GhWRKY42*. **b** Sequence alignment of the deduced *GhWRKY42* protein with its homologous proteins *AtWRKY15* (At2g23320), *OsWRKY17* (XP_015633572), *ZmWRKY21* (XP_008673611), *GmWRKY15* (XP_006573647) and *NtWRKY15* (XP_016498573). The approximately 60-amino acid WRKY domain is indicated by the two-headed arrow. The highly conserved WRKY domain core sequence WRKYGQK is boxed. The C and H residues in the zinc-finger motif are indicated by arrows. The KKRK NLS and conserved HARF structural motif are indicated by a horizontal line. The abbreviations before the gene names of different species are as follows: At, *Arabidopsis thaliana*; Gh, *Gossypium hirsutum*; Zm, *Zea mays*; Gm, *Glycine max*; Os, *Oryza sativa*; Nt, *Nicotiana tabacum*

**Fig. 2:**
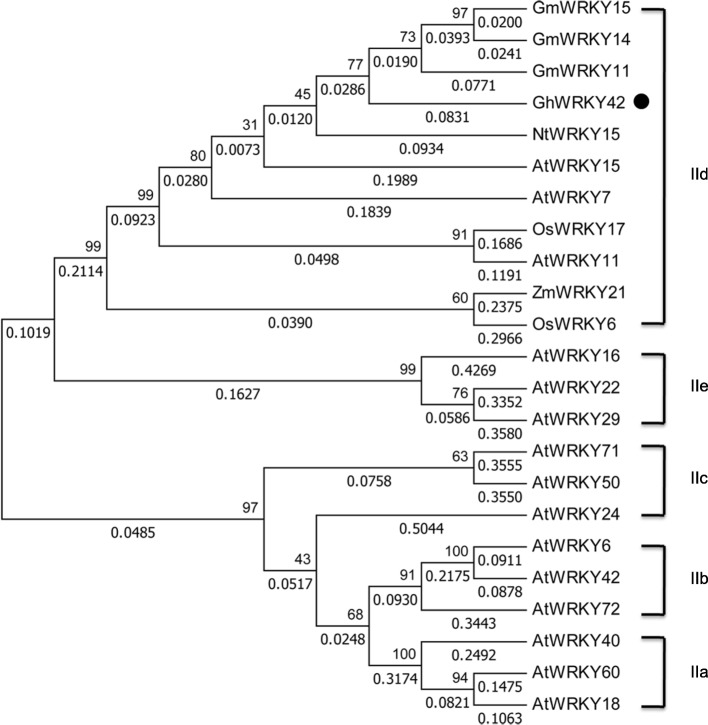
Phylogenetic analyses of *GhWRKY42*. The phylogenetic tree was constructed using the neighbor-joining method in MEGA 7 software. *GhWRKY42* is indicated with a black dot

We previously identified several WRKY genes in cotton that were up regulated by abiotic stresses, during leaf senescence and in vegetative organs using cDNA microarray and RNA-Seq data [23]. Among them, we selected *GhWRKY42* for further study. The sequence analysis results showed that *GhWRKY42* contained a 1038-bp ORF, encoding 345 amino acids. The predicted protein isoelectric point was 9.38, and the molecular weight was 37.88 kDa. The results of comparative analysis of the *GhWRKY42* coding and genomic sequences indicated that *GhWRKY42* harbored three exons and two introns (Fig. 1a). The multiple sequence alignment results revealed that the *GhWRKY42* protein contained one WRKY domain, consisting of a conserved WRKYGQK core sequence and a C2H2 (C-X5-C-X23-H-X1-H) zinc-finger-like motif. Therefore, *GhWRKY42* belongs to the group II WRKY subfamily according to Eulgem et al. [3]. Furthermore, a putative nuclear localization signal (NLS) sequence (KKRK) and a conserved HARF structural motif were found within the *GhWRKY42* amino acid sequence, which are shared among group IId WRKY proteins (Fig. 1b). A phylogenetic tree was built to evaluate the evolutionary relationship between *GhWRKY42* and other group II WRKY members from different species (Fig. 2). As shown in Fig. 2, *GhWRKY42* was closely associated with group IId members, which was consistent with the results of the amino acid alignment analysis.

### *GhWRKY42* localizes to the nucleus

**Fig. 3:**
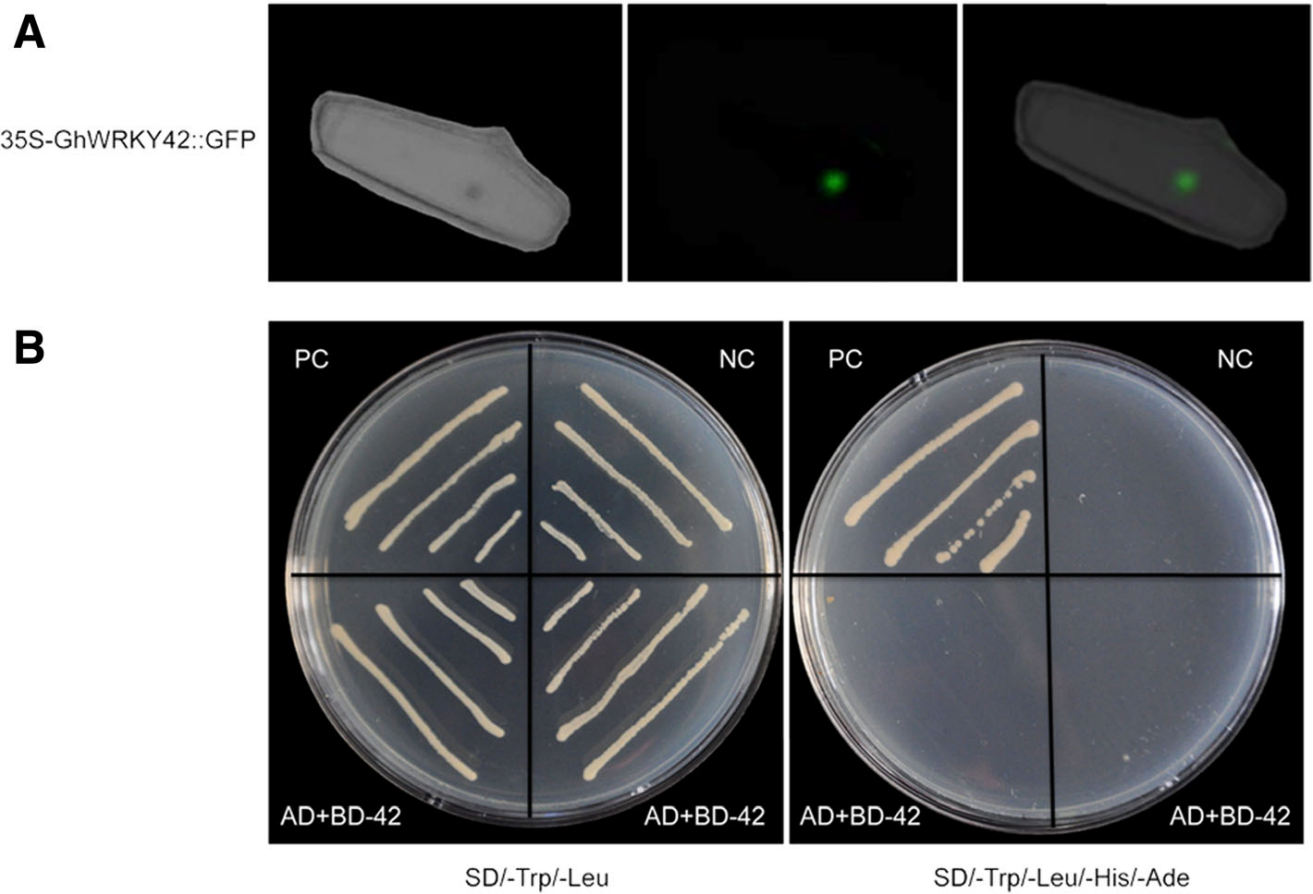
Subcellular localization and transcriptional activity assays of *GhWRKY42*. **a** Transient expression of the *35S-GhWRKY42::GFP* construct in onion epidermal cells. **b** Transcriptional activity of *GhWRKY42* in Y2HGold yeast cells. The ORF of *GhWRKY42* was cloned into the pGBKT7 vector. The constructs were transformed into Y2HGold yeast cells and identified on SD/−Trp/−Leu and SD/−Trp/−Leu/-His/−Ade medium. PC, positive control (pGADT7-largeT+pGBKT7-p53); NC, negative control (pGADT7-largeT+pGBKT7-laminC); AD+BD-42, experimental group (pGADT7-largeT+pGBKT7-*GhWRKY42*)

Consistent with the identified NLS sequence, the subcellular location prediction software Plant-mPloc (http://www.csbio.sjtu.edu.cn/bioinf/plant-multi/) predicted that the *GhWRKY42* protein localizes to the nucleus. To confirm our prediction, the 35S-*GhWRKY42*::GFP vector was constructed and transferred into onion epidermal cells. The 35S::GFP construct served as a control. The onion epidermal cells harboring the 35S-*GhWRKY42*::GFP construct emitted green fluorescence predominantly in nuclei (Fig. 3a), whereas 35S::GFP fluorescence occurred widely throughout the cell [24].

### Transcriptional activation assay of *GhWRKY42*

The transcriptional activation of *GhWRKY42* was examined with a GAL4 yeast system. The plasmids pGADT7-largeT+pGBKT7-*GhWRKY42* (experimental group), pGADT7-largeT+pGBKT7-p53 (positive control) and pGADT7-largeT+pGBKT7-laminC (negative control) were transformed into Y2HGold yeast cells. All transformants grew well on SD/−Trp/−Leu medium. The transformants of the positive control grew well on SD/−Trp/−Leu/-His/−Ade medium, but similar to the negative control, the experimental group did not grow on this medium (Fig. 3b).

### Promoter analysis of *GhWRKY42*

A 1943-bp *GhWRKY42* promoter fragment was obtained, and putative *cis*-elements were analyzed using the PlantCARE database. A group of putative *cis*-elements were identified in the promoter region, which were mainly involved in defense, stress, light and metabolic responses (Additional file 1: Table S1).

**Fig. 4:**
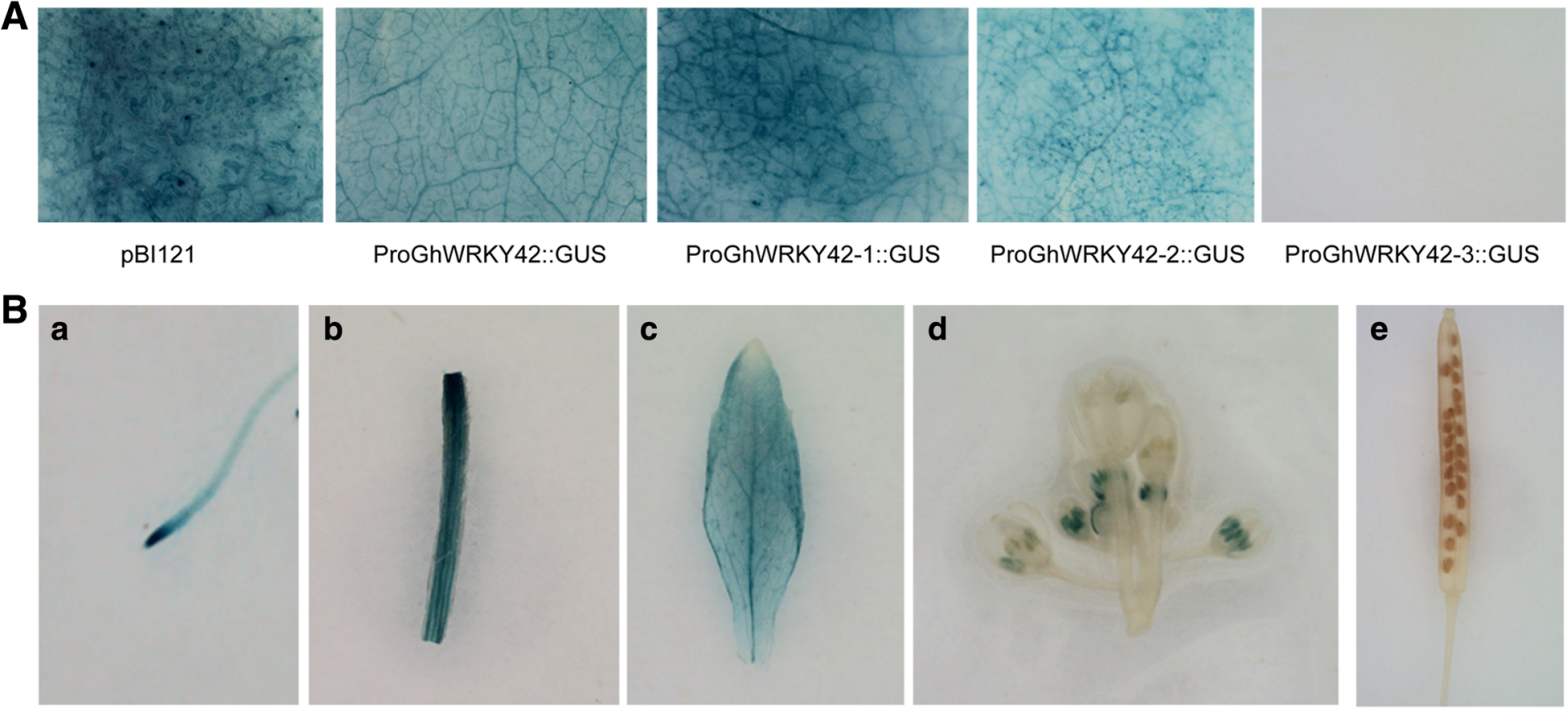
GUS activity analysis of the *GhWRKY42* promoter. **a** Detection of the GUS activity of the *GhWRKY42* promoter deletion vector transiently expressed in *Nicotiana tabacum*. From the left to right, the pBI121 vector (positive control) and the *ProGhWRKY42::GUS* (− 1943 bp to − 1 bp), *ProGhWRKY42–1::GUS* (− 1407 bp to − 1 bp), *ProGhWRKY42–2::GUS* (− 778 bp to − 1 bp) and *ProGhWRKY42–3::GUS* (− 391 bp to − 1 bp) constructs are shown. (**b**) GUS activity of *ProGhWRKY42::GUS* transgenic *Arabidopsis* in different tissues. (a-e) roots, stems, leaves, floral organs and pods

The results of GUS staining for the promoter deletion constructs showed that pBI121 (positive control) as well as *ProGhWRKY42::GUS* (− 1943 bp to − 1 bp), *ProGhWRKY42–1::GUS* (− 1407 bp to − 1 bp) and *ProGhWRKY42–2::GUS* (− 778 bp to − 1 bp) produced blue dots, whereas *ProGhWRKY42–3::GUS* (− 391 bp to − 1 bp) did not (Fig. 4a). The GUS staining results for different tissues showed that GUS was mainly active in the roots, stems and leaves of *ProGhWRKY42::GUS* transgenic *Arabidopsis* plants and was also detectable in the stamens but not in the pistils, petals or pods (Fig. 4b).

### Expression analysis of *GhWRKY42* under stress treatments

**Fig. 5:**
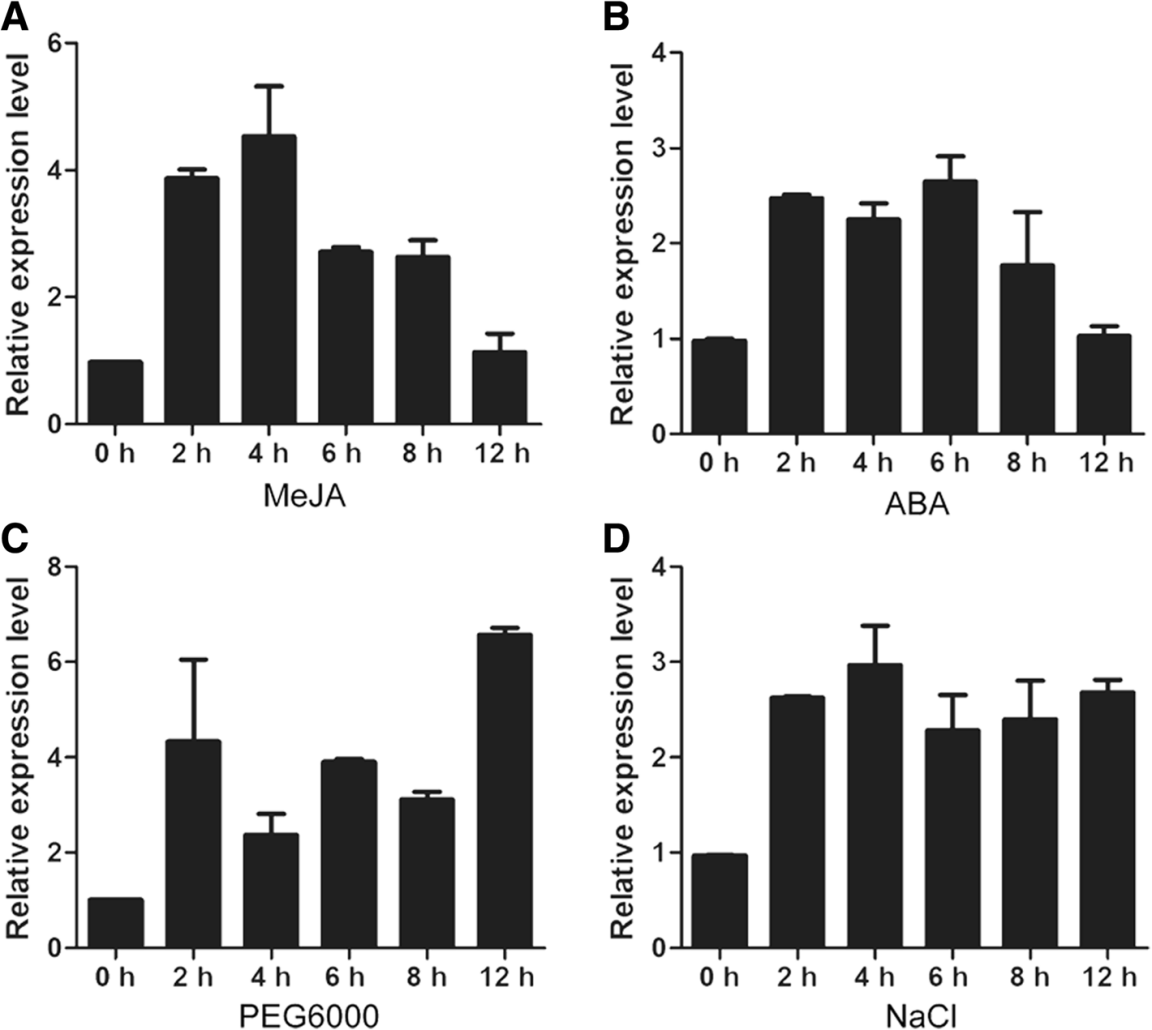
Expression profiles of *GhWRKY42* under different abiotic stresses. Ten-day-old cotton seedlings were exposed to (**a**) 100 μM MeJA, (**b**) 200 μM ABA, (**c**) 15% PEG6000 and (**d**) 200 mM NaCl. Samples were collected at 0 h, 2 h, 4 h, 6 h, 8 h and 12 h. Total RNA was extracted for qRT-PCR. *GhActin* served as the reference gene. The bars represent the means ± standard errors (SEs) from three independent experiments

To evaluate the expression patterns of *GhWRKY42* following various stresses, ten-day-old cotton seedlings were exposed to MeJA, ABA, drought and salt treatments. As shown in Fig. 5, *GhWRKY42* was found to be differentially up-regulated under MeJA, ABA, drought and salt treatments. *GhWRKY42* expression was rapidly induced at 2 h after MeJA treatment, reaching its maximum accumulation at 4 h (4.6-fold induction) and then gradually decreasing (Fig. 5a). Similarly, *GhWRKY42* expression was induced at 2 h after ABA treatment but exhibited maximum transcript levels at 6 h with 2.7-fold induction (Fig. 5b). Under drought treatment, the *GhWRKY42* transcript was differently elevated at different time points and peaked at 12 h (6.5-fold induction) (Fig. 5c). However, under salt treatment, the expression of *GhWRKY42* was dramatically increased at 2 h, and a high expression level was maintained in the subsequent 4–12 h (Fig. 5d).

### Expression analysis of *GhWRKY42* in different tissues and during leaf senescence

**Fig. 6:**
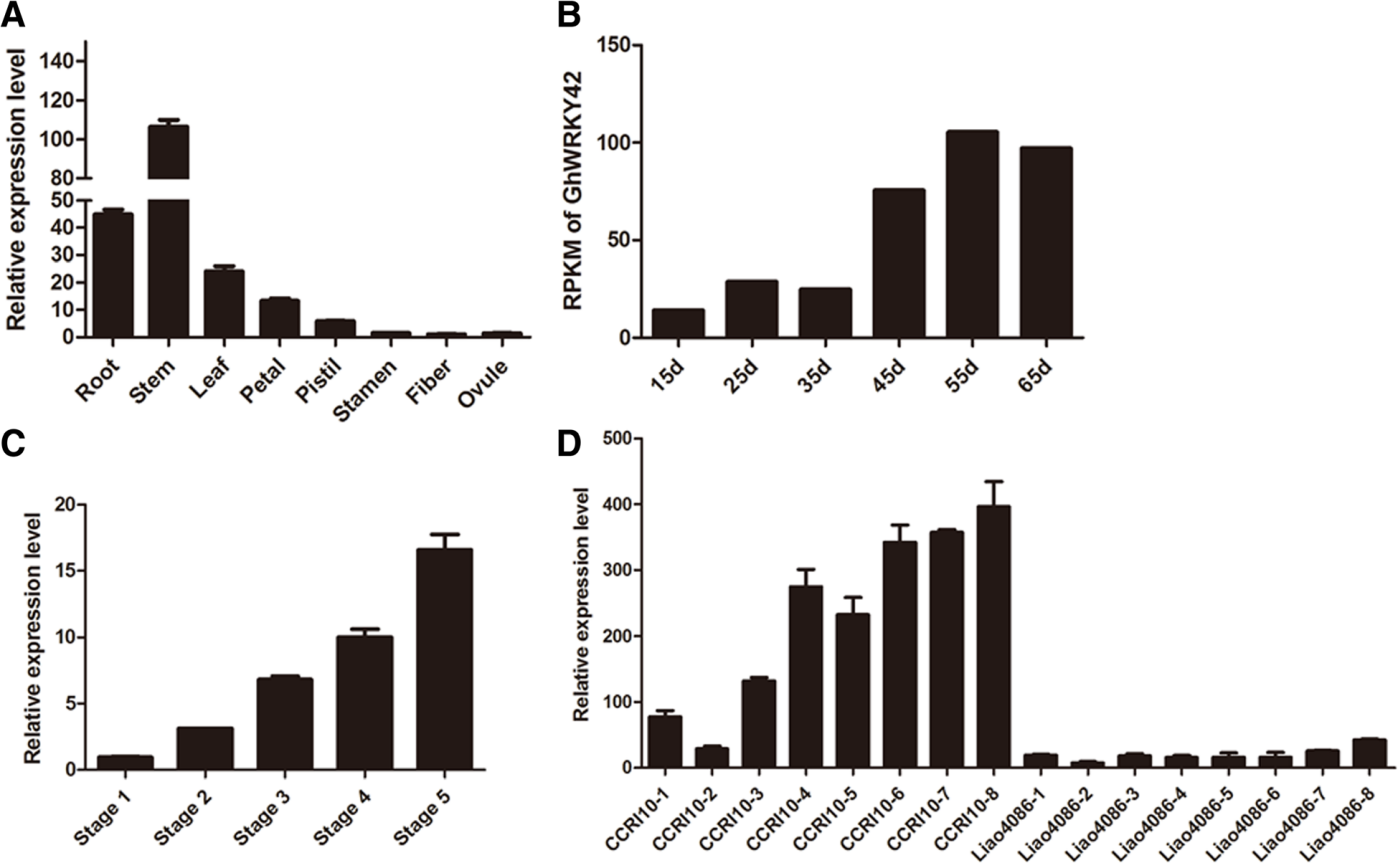
Expression profiles of *GhWRKY42* in different tissues and during leaf senescence in cotton. **a** Expression levels of *GhWRKY42* in different tissues. **b** Expression profile of *GhWRKY42* during leaf senescence in 15-, 25-, 35-, 45-, 55-, and 65-day-old leaves. RPKM, reads per kilobase per million mapped reads. **c** Expression level of *GhWRKY42* in true leaves at different developmental stages. Stage 1, a fully expanded, young, non-senescent leaf; Stage 2, a fully expanded, mature, non-senescent leaf; Stage 3, a leaf in early senescence with < 25% leaf area yellowing; Stage 4, a leaf in mid-senescence with approximately 50% leaf area yellowing; Stage 5, a leaf in late senescence with > 75% leaf area yellowing. **d** Relative expression level of *GhWRKY42* in the cotyledons of the CCRI 10 and Liao 4086 varieties. Cotyledon samples were collected weekly at eight different development stages, ranging from the flattened cotyledon stage to the completely aged stage. *GhActin* served as the reference gene. The bars represent the means ± SEs from three independent experiments

qRT-PCR was performed to detect the transcript levels of *GhWRKY42* in the roots, stems, leaves, petals, pistils, stamens, fiber and ovules. *GhWRKY42* was found to be differentially expressed in different tissues. *GhWRKY42* was strongly expressed in vegetative organs, including the stems, roots and leaves but was weakly expressed in the petals, pistils, stamens, fiber and ovules (Fig. 6a).

To evaluate the expression pattern of *GhWRKY42* during leaf senescence, qRT-PCR was performed using cotton leaves at different senescence stages. The transcriptome data analysis [23, 25] showed that the expression level of *GhWRKY42* gradually increased with the senescence of leaves (Fig. 6b). We further examined the expression level of *GhWRKY42* in true leaves of CCRI74 plants at different senescence stages [24]; the results revealed that the transcript levels of *GhWRKY42* gradually increased as the leaves aged (Fig. 6c). In addition, the expression level of *GhWRKY42* was detected in cotyledon samples from the early-aging cotton variety CCRI10 and the non-early-aging variety Liao4086. The qRT-PCR results showed that the transcript levels of *GhWRKY42* increased gradually during cotyledon senescence and were significantly higher in CCRI10 than in Liao4086 (Fig. 6d).

### *GhWRKY42* promotes leaf senescence in transgenic *Arabidopsis*

**Fig. 7:**
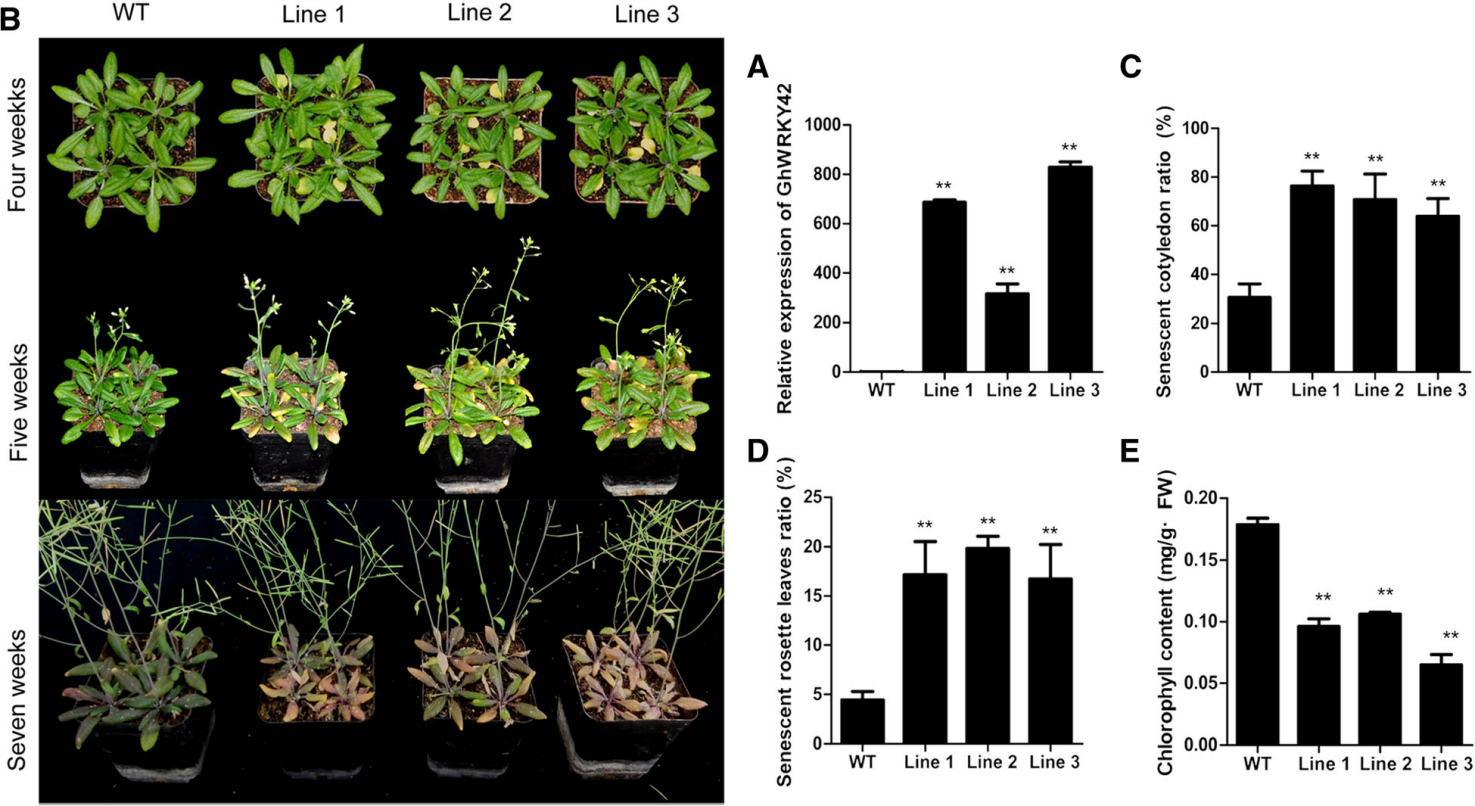
Overexpression of *GhWRKY42* in *Arabidopsis* promotes leaf senescence. **a** Transcript levels of *GhWRKY42* in WT and transgenic lines. **b** Phenotypes of WT and transgenic lines grown for four, five and seven weeks. **c** Relative senescent cotyledon ratio of the WT and transgenic lines after four weeks. **d** Relative senescent true leaf (rosette leaves) ratio of the WT and transgenic lines after five weeks. **e** Chlorophyll contents of the WT and transgenic lines after seven weeks. *AtActin2* served as the reference gene. The bars represent the means ± SEs from three independent experiments. Independent t-tests revealed highly significant (**P < 0.01) differences between the WT and the transgenic lines

The transcript of *GhWRKY42* was highly accumulated in the senescent leaves of cotton. To further clarify the functional role of *GhWRKY42* in response to leaf senescence, *GhWRKY42* was transformed into *Arabidopsis* plants. The transgenic lines were confirmed by qRT-PCR (Fig. 7a). As shown in Table 1, the *GhWRKY42* transgenic plants flowered earlier and had fewer rosette leaves than the WT plants. In addition, the senescence phenotypes of the transgenic and WT plants were observed at different developmental stages, and the ratio of senescent leaves was counted. Compared with the WT, the transgenic lines exhibited severe aging phenotypes at four, five and seven weeks (Fig. 7b), which were reflected by a significantly higher ratio of senescent cotyledons at four weeks (Fig. 7c), a higher ratio of senescent true leaves (rosette leaves) at five weeks (Fig. 7d) and a lower chlorophyll content at seven weeks (Fig. 7e).

**Table 1:** Comparison of flowering time and rosette leaf numbers

Genotype^a^	Anthesis(DAS)^b^	Number of rosette leaves	N
WT	31.86 ± 0.99	12.53 ± 1.48	36
Line 1	30.78 ± 1.29^**^	11.67 ± 1.41^*^	36
Line 2	31.03 ± 1.38^*^	11.86 ± 1.40	36
Line 3	30.61 ± 1.34^**^	11.25 ± 1.5^**^	36

Plants were grown under long days (16 h/8 h)

*Values significantly differ from those of WT at the 0.05 confidence level

**Values significantly differ from those of WT at the 0.01 the confidence level

^a^Genetic background: Columbia; transgenic lines of *GhWRKY42*

^b^Indicators of anthesis [days after sowing (DAS)] are shown as the means ± standard deviation (SD)

N represents the number of plants used for statistics

### Increased expression levels of senescence-associated marker genes and ABA-responsive genes in *GhWRKY42*-overexpressing plants

**Fig. 8:**
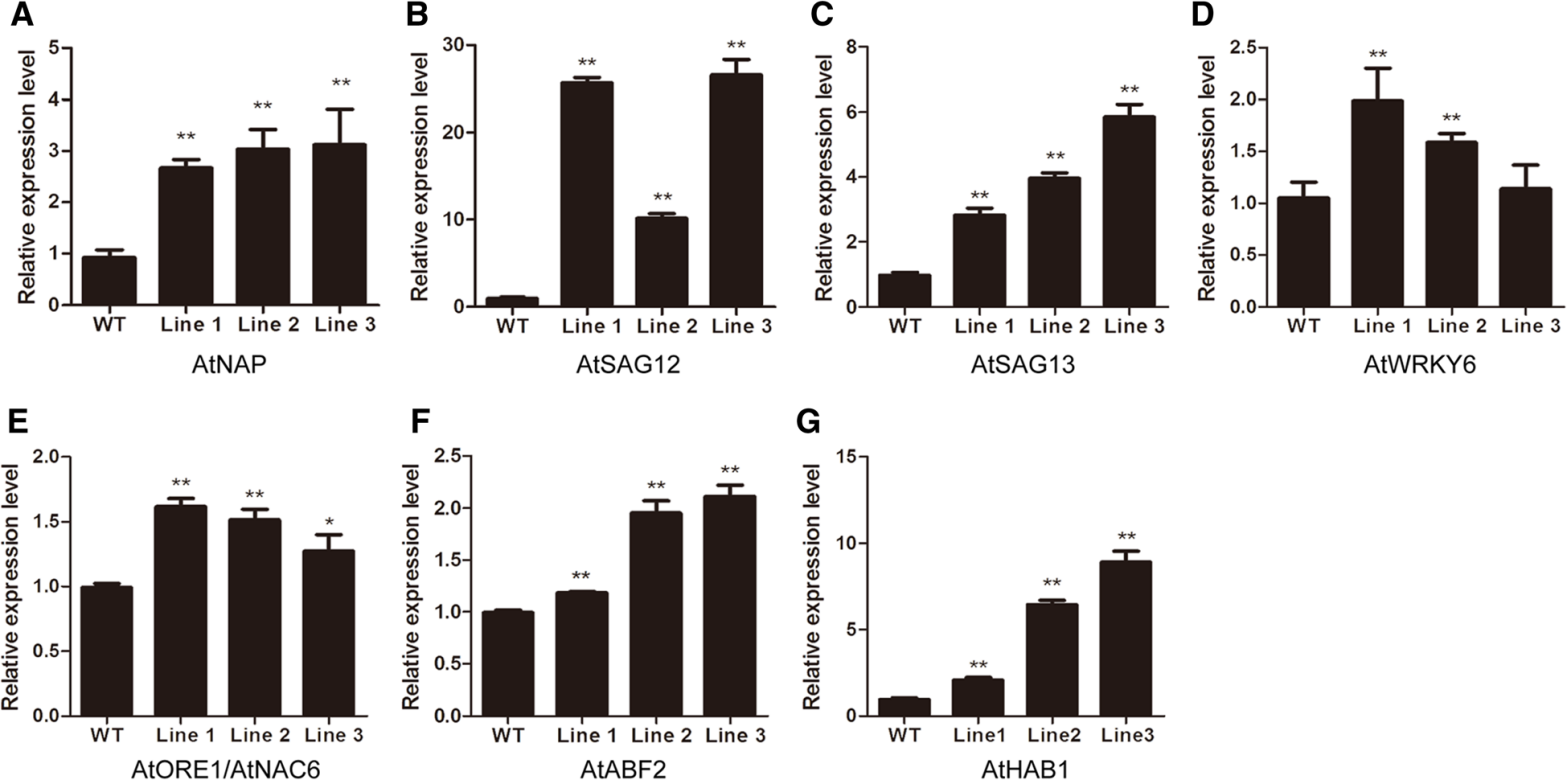
Expression levels of SAGs and ABA-responsive genes in WT and transgenic plants during leaf senescence. E**x**pression levels of *AtNAP* (**a**), *AtSAG12* (**b**), *AtSAG13* (**c**), *AtWRKY6* (**d**), *AtORE1*/*AtNAC6* (**e**), *AtABF2* (**f**) and *AtHAB1* (**g**) in WT and transgenic plants. The *AtNAP*, *AtSAG12*, *AtSAG13*, *AtWRKY6* and *AtORE1*/*AtNAC6* genes are SAGs. The *AtABF2* and *AtHAB1* genes are ABA-responsive genes. Total RNA was isolated from five-week-old *Arabidopsis* rosette leaves. *GhActin* served as the reference gene. The bars represent the means ± SEs from three independent experiments. Independent t-tests revealed highly significant (**P < 0.01) differences between the WT and the transgenic lines

To elucidate the possible mechanisms of *GhWRKY42*-mediated precocious senescence, we examined the effects of *GhWRKY42* on the transcript levels of senescence-associated marker genes during natural leaf senescence. The genes included *AtNAP* (NAC domain TF) (At1g69490), *AtSAG12* (At5g45890), *AtSAG13* (At2g29350), *AtWRKY6* (WRKY DNA-binding protein 6) (At1g62300) and *AtORE1*/*AtNAC6* (NAC domain TF) (At5g39610), which are all factors that are up-regulated during aging in *Arabidopsis* [26–30]. As shown in Fig. 8a-e, the expression of all senescence-associated marker genes in the transgenic plants was significantly up-regulated compared with that in the WT plants. In addition, we identified the expression levels of two ABA-responsive genes, *AtABF2* (ABA-responsive element binding factor 2) (AT1G45249) [31] and *AtHAB1* (hypersensitive to ABA1) (AT1G71770) [32], in *Arabidopsis*. The expression levels of both genes were significantly elevated in the transgenic plants compared with the WT plants (Fig. 8f, g).

### Decreased plant height in *GhWRKY42*-silenced cotton plants obtained via VIGS

**Fig. 9:**
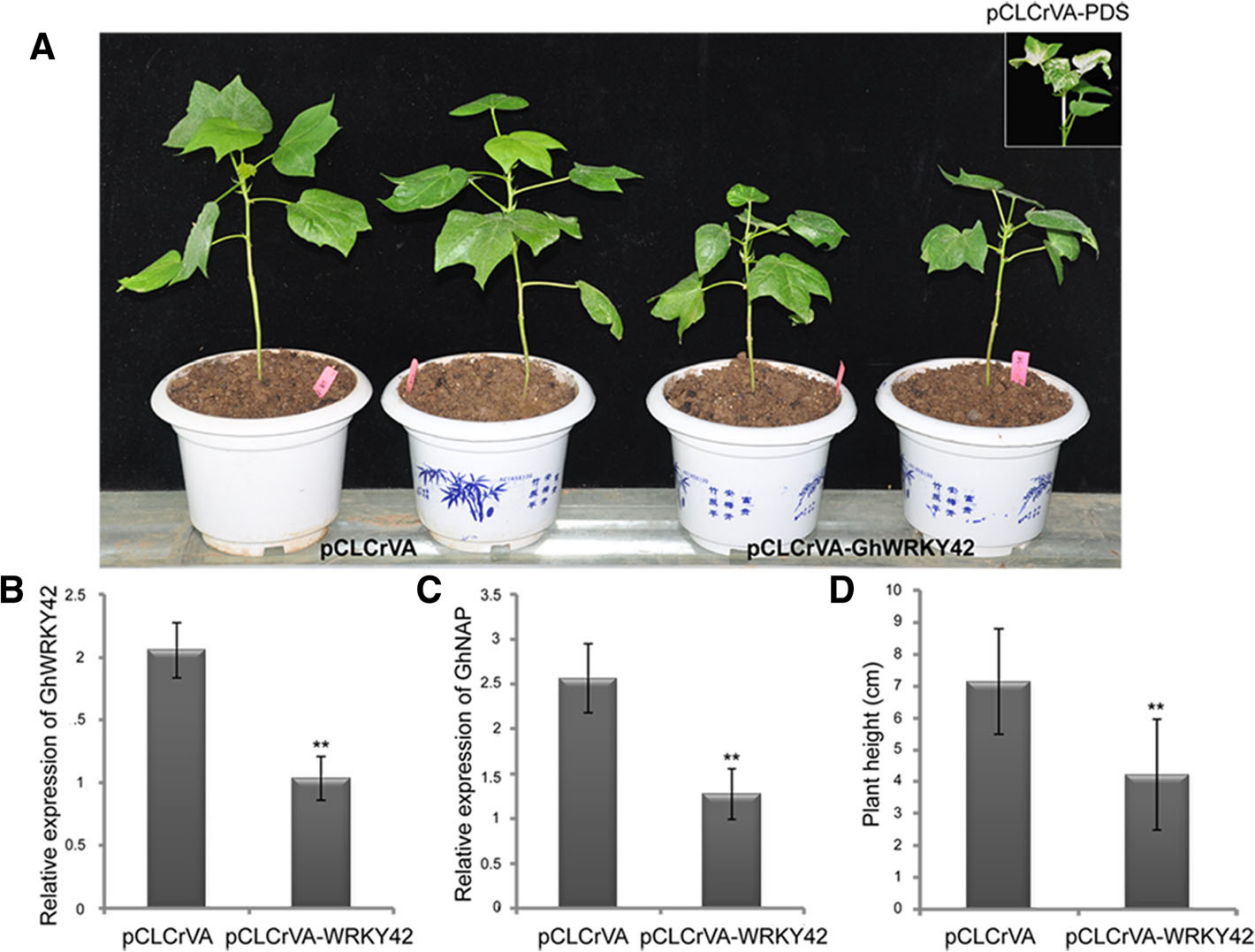
Silencing *GhWRKY42* via VIGS decreases plant height in cotton. **a** Plant phenotypes of empty control and *GhWRKY42***-**silenced plants. **b** Expression level of *GhWRKY42* in empty control and *GhWRKY42*-silenced plants. **c** Expression level of *GhNAP* in empty control and *GhWRKY42*-silenced plants. (**d**) Plant height of empty control and *GhWRKY42*-silenced plants. *GhActin* served as the reference gene. The bars represent the means ± SEs from three independent experiments. Independent t-tests revealed highly significant (**P < 0.01) differences between the control and silenced plants

To further identify the functional role of *GhWRKY42*, VIGS of *GhWRKY42* was performed using the cotton variety CCRI10. Two weeks later, the cotton plants harboring pCLCrVA-PDS showed an albino phenotype, suggesting that the VIGS assay was successful (Fig. 9a). qRT-PCR was performed to evaluate the effect of gene silencing. Expression level of *GhWRKY42* was significantly lower in the silenced plants (pCLCrVA-*GhWRKY42*) than in the control plants (pCLCrVA) (Fig. 9b). The expression of the senescence-associated marker gene *GhNAP* was also markedly reduced in the silenced plants (Fig. 9c). As shown in Fig. 9a, the silenced plants exhibited a relatively lower plant height phenotype than the control plants, and the lower plant height phenotype was statistically analyzed (Fig. 9d).

## Discussion

The WRKY TF family is one of the largest superfamilies of regulatory proteins in plants [7]. In the past several years, growing evidence has shown that members of the WRKY gene family mainly participate in stress responses, plant growth and development and leaf senescence [33]. However, studies on WRKY TFs have mainly focused on model plant species, while only a few of these genes have been evaluated in cotton. In this study, we isolated a group IId *GhWRKY42* gene from upland cotton and characterized its functional roles. The results of multiple sequence alignment and phylogenetic tree analyses revealed that the *GhWRKY42* gene is a member of the group IId WRKY family. Subcellular localization analysis revealed that the *GhWRKY42* protein is located in the nucleus. These findings are consistent with the predicted nuclear-targeting signal sequence and with the results of studies on another group IId TF, *GhWRKY11*, in cotton [34]. The results of transcriptional activation analysis in yeast have shown that the *GhWRKY42* protein has no transcriptional activation activity; these findings are similar to those reported for *PtrWRKY40* from *Populus trichocarpa* [35]. These results suggest that *GhWRKY42* may be a nuclear protein that functions in the cell nucleus but may be not a transcriptional activator.

The expression patterns of genes are often used as an indicator of their functional roles [36]. For example, *GhWRKY17* has been shown to be induced by salt and drought treatments, and overexpression of *GhWRKY17* in *N. tabacum* results in a more sensitive phenotype to drought and salt stresses [37]. Previous studies have shown that a large number of genes can be induced by various abiotic stresses [38]. In our study, *GhWRKY42* was demonstrated to be differentially induced under MeJA, ABA, drought and salt treatments in cotton. In addition, many stress response *cis*-elements were found in the promoter region of *GhWRKY42*. These findings suggested that *GhWRKY42* might be involved in the regulation of abiotic stress networks.

The 5′ promoter deletion assay is often used to investigate promoter expression characteristics and the functional roles of regulatory elements in promoter regions. The structure and function of promoter deletion fragments can be suggested by evaluating promoter deletion construct-driven reporter genes in transgenic plants [39]. In our study, a promoter deletion assay showed that *ProGhWRKY42–3::GUS* (− 391 bp to − 1 bp) was unable to activate expression of the GUS gene and that *ProGhWRKY42–2:GUS* (− 778 bp to − 1 bp) contained the shortest sequence exhibiting promoter activity. Therefore, it is speculated that critical *cis*-elements may exist within the − 778 bp to − 391 bp upstream region of the *GhWRKY42* promoter. The *cis*-elements in this region were predicted, it was found to contain not only TATA-box and light response elements but also stress response elements such as ABRE (ABA response element), the CGTCA motif (MeJA response element), HSE (heat response element) and the TGACG motif (MeJA response element). These elements may play an important role in ensuring that the promoter drives the expression of downstream genes.

WRKYs can directly bind to the W-box [TTGAC(C/T)] in the promoter of target genes to modulate stress responses, plant development and leaf senescence [40, 41]. Liu et al. reported that W-box and G-box *cis*-elements are important positive regulators during leaf senescence in rice. Both elements are significantly plentiful in the promoter regions of up-regulated TFs (including WRKYs) that regulate leaf senescence [41]. W-box and G-box *cis*-elements were identified in the promoter region of *GhWRKY42*, suggesting that *GhWRKY42* may be involved in leaf senescence and be regulated by other *GhWRKY*s or by the gene itself during this process. These findings laid the foundation for further analysis of the upstream regulatory mechanism of *GhWRKY42*.

Senescence is a natural phenomenon and prevails among all living organisms, including plants. During leaf senescence, genetic and environmental factors affect mature leaves, leading to the initiation of leaf senescence; this senescence is accompanied by chlorophyll, membrane, protein and nucleic acid degradation as well as nutrient relocation from senescing leaves to growing organs or storage tissues [42–45]. Crop productivity is mainly determined by the yield per area, but leaf senescence severely affects crop yield [46]. Thus, studying the mechanism of leaf senescence is particularly important. In the present study, the expression level of *GhWRKY42* was found to be up-regulated during natural senescence and exhibited significantly higher expression in the early-aging cotton variety CCRI10 than in the non-early-aging variety Liao4086. It has been reported that the *GhNAC12* gene, which is more highly expressed in CCRI10 than in Liao4086 during leaf senescence, causes an early-aging phenotype in *Arabidopsis* [47]. Therefore, *GhWRKY42* may be involved in the aging process and may play a positive regulatory role during leaf senescence. Consistent with our prediction, overexpression of *GhWRKY42* did lead to an advance of leaf senescence in transgenic *Arabidopsis*. In a previous study, overexpression of *AtWRKY45* in *Arabidopsis* was observed to up-regulate expression of representative SAGs during age-triggered leaf senescence [13]. Phenotypic observations of overexpressing *Arabidopsis* lines and RNAi cotton lines show that *GhNAP* positively regulates leaf senescence through ABA-mediated pathways [48]. Phytohormones, such as ABA, ethylene, MeJA, and salicylic acid, have been demonstrated to promote leaf senescence [45]. The ABA content increases in aged leaves, and endogenously applied ABA promotes expression of some SAGs [44]. ABA-responsive genes, which are involved in the ABA signaling pathway, are induced in senescing *Arabidopsis* [49]. In our study, SAGs and ABA-responsive genes were significantly accumulated in the *GhWRKY42* transgenic lines, suggesting that *GhWRKY42* may be associated with leaf senescence via ABA-mediated pathways.

Previous studies have shown that some genes are closely associated with plant height. Wei et al. identified the QTL DTH8 in rice, which includes the HAP3 gene and regulates yield, plant height and flowering time [50]. WRKY TFs such as *LP1* in foxtail millet and *OsWRKY78* in rice have all been shown to play an important role in stem elongation and plant height [51, 52]. In our study, we detected high expression levels and strong GUS activity of *GhWRKY42* in the stem and reduced height in VIGS plants. Therefore, we hypothesized that *GhWRKY42* might be related to stem development. Plant height is an important plant architecture trait, and decreased height is beneficial for mechanical harvesting and lodging resistance [53]. Our findings provide a basis for breeding new cotton varieties with an ideal plant type. However, further studies are needed to elucidate the pathways involved in the *GhWRKY42*-mediated mechanism.

## Conclusions

*GhWRKY42*, a group IId WRKY member, is closely associated with leaf senescence and plant development. *GhWRKY42* is located in the nucleus and exhibits no transcriptional activity. *GhWRKY42* is induced by leaf senescence and various stresses. Ectopic expression of *GhWRKY42* in *Arabidopsis* promotes leaf senescence, and VIGS cotton plants exhibit a decreased plant height phenotype. Our work could lead to a better understanding of the functional roles of WRKY genes in cotton. However, how the *GhWRKY42* gene regulates leaf senescence and plant height development requires further study and clarification.

## Methods

### Plant materials, growth conditions and stress treatments

Two early-aging cotton varieties, CCRI10 and CCRI74, and a non-early-aging variety, Liao4086, were used in our experiments. The cotton varieties were cultivated in the field of the Cotton Research Institute of the Chinese Academy of Agricultural Sciences (Anyang, Henan, China). Different tissues were collected from CCRI10 plants. Roots and stems were collected from two-week-old seedlings. Leaves were collected from newly flattened leaves. Petals, pistils and stamens were sampled at anthesis, and fiber and ovules were harvested at 10 days post anthesis.

To evaluate the expression pattern of *GhWRKY42* during leaf senescence, cotyledons were collected from two cotton varieties, CCRI10 and Liao4086, which exhibit different aging characteristics. We collected cotyledon samples weekly at eight different developmental stages, ranging from the flattened cotyledon stage to the completely aged stage. The expression patterns of *GhWRKY42* were further evaluated in true leaves of the early-aging cotton variety CCRI74 at five aging stages, as described previously [24]. Each sample included material from eight different individual plants, and we performed three repetitions for each sample.

To evaluate the stress response of *GhWRKY42* in cotton, 10-day-old CCRI10 cotton seedlings were planted in pots for subsequent stress treatments. The CCRI10 cotton seedlings were planted in a growth chamber at 25 °C, with a 16 h light/8 h dark cycle. For the abiotic stress treatment, the seedlings were irrigated with 15% polyethylene glycol 6000 (PEG6000) and 200 mM sodium chloride (NaCl); for the signaling molecule treatment, the seedlings were sprayed with 100 μM methyl jasmonate (MeJA) and 200 μM abscisic acid (ABA). Each cotyledon sample included material collected from eight uniform plants, and each treatment was repeated three times. The samples were harvested at 0 h, 2 h, 4 h, 6 h, 8 h and 12 h. All samples were quickly frozen in liquid nitrogen for subsequent RNA extraction.

### Gene cloning and sequence analysis

To amplify the full-length cDNA and genomic sequences of *GhWRKY42*, primers were designed based on the coding sequence of *GhWRKY42* (accession KF669797) submitted to NCBI by Dou et al. [23]. The primers used for this purpose are listed in Additional file 2: Table S2. The full-length cDNA and genomic fragments of *GhWRKY42* were amplified from cDNA and DNA, respectively, obtained from CCRI10 leaves at the five-leaf stage. The fragments were subsequently inserted into the pMD18-T vector (TaKaRa, China) and transformed into *Escherichia coli* competent cells (*E. coli* DH5a) for sequencing. The genomic and coding sequences of *GhWRKY42* were submitted to Gene Structure Display Server online software (GSDS2.0) (http://gsds.cbi.pku.edu.cn/) to predict gene structures. Multiple sequence alignment was conducted using DNAMAN software, and a phylogenetic tree was built by using MEGA 7 software. The *GhWRKY42* promoter fragment was amplified from DNA, and the online software PlantCARE (http://bioinformatics.psb.ugent.be/webtools/plantcare/html/) was employed to predict *cis*-acting elements.

### RNA isolation, cDNA synthesis and qRT-PCR

Total RNA was isolated using RNAprep PurePlant Kit (Polysaccharides & Polyphenolics-rich) (Tiangen, China). One microgram of total RNA was prepared for cDNA synthesis in a 20 μl reaction system using a PrimeScript™ RT reagent kit with gDNA Eraser. The cDNA was diluted 5 times for qRT-PCR. Transcript levels were detected using a 7500 Real-Time PCR system (Applied Biosystems) and SYBR® Premix Ex Taq™ II (Tli RNaseH Plus) (TaKaRa). The 20 μl reaction volume contained the following components: 10 μl of SYBR Premix Ex Taq II (Tli RNaseH Plus) (2×), 0.8 μl of the PCR forward primer (10 μM), 0.8 μl of the PCR reverse primer (10 μM), 0.4 μl of ROX Reference Dye II (50×), 2 μl of cDNA and 6 μl of ddH_2_O. The optimal PCR amplification procedure used was as follows: a pre-denaturation step at 95 °C for 30 s; 40 cycles of 95 °C for 5 s and 60 °C for 34 s; and a melting curve step at 95 °C for 15 s, 60 °C for 1 min and 95 °C for 15 s. *GhActin* and *AtActin2* were used as reference genes. The 2^−ΔΔCT^ method was applied to calculate relative expression levels [54]. Three independent experiments were performed, and all reactions were performed with three technical replicates.

### Subcellular localization

The open reading frame (ORF) of *GhWRKY42* without the termination codon was cloned into the pBI121-GFP vector to generate the 35S-*GhWRKY42*::GFP construct, driven by the *cauliflower mosaic virus* 35S promoter. The 35S-*GhWRKY42*::GFP plasmid was extracted to obtain a plasmid concentration of at least 1 μg/μl. The inner epidermis of a fresh onion was cut into approximately 1.5 × 1.5 cm pieces with a scalpel on a clean bench. The epidermal pieces were then transferred to solid Murashige and Skoog (MS) medium and cultivated at 28 °C for 3–6 h in darkness. The gene gun device was sterilized and was placed on a clean bench, and the bombarding chamber and some accessories were cleaned with 75% alcohol. After the particulate carrier membrane was washed with 70 and 100% alcohol, plasmids encased in gold powder were added to the middle of the particulate carrier membrane. After the membrane dried slightly, the onion epidermis was bombarded using the gene gun with the following parameters: particle bombardment running distance, 9 cm; rupture disk pressure, 1300 psi; and vacuum degree, 28 mmHg. The epidermis after bombardment was transferred to fresh MS agar medium at 25 °C for 12 h in darkness. The resulting green fluorescence was detected using a confocal laser scanning microscope (Zeiss LSM 700) at a wavelength of 488 nm.

### Transcriptional activation assays

The ORF of *GhWRKY42* was cloned into the pGBKT7 vector to construct pGBKT7-*GhWRKY42*. The pGADT7-largeT+pGBKT7-*GhWRKY42* (experimental group), pGADT7-largeT+pGBKT7-p53 (positive control) and pGADT7-largeT+pGBKT7-laminC (negative control) plasmids were transformed into Y2HGold yeast competent yeast cells. The transformed yeast products were spread on corresponding dropout selective medium plates that did not contain tryptophan or leucine (SD/−Trp/−Leu) and incubated for 3–5 days at 30 °C. Positive clones were identified and streaked on SD/−Trp/−Leu medium plates and plates containing medium without tryptophan, leucine, histidine or adenine (SD/−Trp/−Leu/-His/−Ade). The plates were inverted and incubated at 30 °C for 3–5 days to identify transcriptional activity.

### Genetic transformation of *Arabidopsis thaliana*

The ORF of *GhWRKY42* was inserted into the binary expression vector pBI121 driven by the 35S promoter to generate the *35S::GhWRKY42* construct*.* The *GhWRKY42* promoter fragment was also inserted into the pBI121 vector by replacing the 35S promoter to generate the *ProGhWRKY42::GUS* construct. The *35S::GhWRKY42* and *ProGhWRKY42::GUS* constructs were individually introduced into *Agrobacterium tumefaciens* strain LBA4404 and transformed into Arabidopsis ecotype Columbia using the floral-dip method [55]. For the screening of positive plants, seeds of the T0 generation (harvested from the wild-type (WT)) were sterilized and selected on 1/2 MS solid medium plates (0.22% MS modified basal salt mixture, 3% sucrose and 0.8% agar powder) containing kanamycin (50 mg/L). The plates containing the seeds were chilled at 4 °C for 3 days in darkness, after which they were transferred to an incubator at 22 °C under a 16 h light/8 h dark cycle with a light intensity of 100 μmol m^− 2^ s^− 1^. Two weeks later, the green seedlings on the plates were selected and transplanted into the nutrient soil in a growth chamber. The positive plants were further verified using PCR, and selfed seeds harvested from the positive plants were employed as the T1 generation. Using the same method, the seeds were screened until the T3 homozygous generation. The phenotypic characteristics of the transgenic and WT plants were observed at different developmental stages.

### Transient transformation of *N. tabacum* and GUS histochemical staining assays

Based on the position of stress response *cis*-elements in the *GhWRKY42* promoter, four promoter deletion fragments were delimited. The four fragments were amplified from the pMD18-T vector containing the *GhWRKY42* promoter and inserted into the pBI121 vector by replacing the 35S promoter. As a result, four promoter deletion plasmids, *ProGhWRKY42::GUS* (− 1943 bp to − 1 bp), *ProGhWRKY42–1::GUS* (− 1407 bp to − 1 bp), *ProGhWRKY42–2::GUS* (− 778 bp to − 1 bp) and *ProGhWRKY42–3::GUS* (− 391 bp to − 1 bp), were constructed and transformed into LBA4404. Transient expression in *N. tabacum* was performed in accordance with previously described methods [56]. Transgenic *Arabidopsis* plants harboring the *ProGhWRKY42::GUS* construct were used to analyze organizational expression characteristics. GUS staining was performed as follows: the prepared materials were soaked in the GUS dye solution, after which the materials were placed in darkness at 25–37 °C overnight; the materials were then decolorized approximately 2–3 times using 70% alcohol until the negative control materials turned white, and the blue dots in the white background observed under microscopy were identified as GUS expression sites.

### VIGS assay

For the VIGS assay, approximately 300-bp fragments amplified from the pMD18-T vector containing the *GhWRKY42* gene were integrated into the pCLCrVA vector to construct pCLCrVA*-GhWRKY42*, which was then transformed into LBA4404. The LBA4404 strains carrying pCLCrVA-*GhWRKY42*, pCLCrVA (negative control) or pCLCrVA-PDS (positive control) were mixed with the strain harboring pCLCrVB (helper vector) (1:1 ratio, OD600 = 1.5) and co-injected into two fully expanded cotyledons of CCRI10 plants. In the VIGS assay, at least 20 seedlings were used per group. For qRT-PCR detection, samples from at least 6 uniform injected plants were used. The cotton plants were then cultivated at 22 °C with a 16 h light/8 h dark cycle in a greenhouse. The experiment was repeated three times. The detailed VIGS procedure was performed as previously described [57, 58].

### Determination of chlorophyll content

Determination of the chlorophyll content was performed as described by Shah et al. [59].

## Additional files

Additional file 1: **Table S1**. Predicted *cis*-acting elements in the promoter region of *GhWRKY42. (DOCX 36 kb)*

Additional file 2: **Table S2**. Primers used in this study. (DOCX 32 kb)

## Abbreviations

ABA: Abscisic acid
DAS: Days after sowing
GUS: β-glucuronidase
MeJA: Methyl jasmonate
MS: Murashige and Skoog
NaCl: Sodium chloride
NLS: Nuclear localization signal
ORF: Open reading frame
PEG6000: Polyethylene glycol 6000
qRT-PCR: Quantitative real-time PCR
SAGs: Senescence-associated genes
SEs: Standard errors
TFs: Transcription factors
VIGS: Virus-induced gene silencing
WT: Wild- type

## References

[1] Tuteja N (2007). Abscisic acid and abiotic stress signaling. Plant Signal Behav.

[2] Ulker B, Somssich IE (2004). WRKY transcription factors: from DNA binding towards biological function. Curr Opin Plant Biol.

[3] Eulgem T, Rushton PJ, Robatzek S, Somssich IE (2000). The WRKY superfamily of plant transcription factors. Trends Plant Sci.

[4] Xie Z, Zhang ZL, Zou XL, Huang J, Ruas P, Thompson D, Shen QJ (2005). Annotations and functional analyses of the rice WRKY gene superfamily reveal positive and negative regulators of abscisic acid signaling in aleurone cells. Plant Physiol.

[5] Rinerson CI, Rabara RC, Tripathi P, Shen QXJ, Rushton PJ. The evolution of WRKY transcription factors. BMC Plant Biol. 2015;15:66. 10.1186/s12870-015-0456-y PMC4350883 25849216

[6] Rushton DL, Tripathi P, Rabara RC, Lin J, Ringler P, Boken AK, Langum TJ, Smidt L, Boomsma DD, Emme NJ *et al*: WRKY transcription factors: key components in abscisic acid signalling. Plant Biotechnol J 2012, 10(1):2–11. 10.1111/j.1467-7652.2011.00634.x 21696534

[7] Rushton PJ, Somssich IE, Ringler P, Shen QXJ (2010). WRKY transcription factors. Trends Plant Sci.

[8] Yamamoto S, Nakano T, Suzuki K, Shinshi H (2004). Elicitor-induced activation of transcription via W box-related cis-acting elements from a basic chitinase gene by WRKY transcription factors in tobacco. Biochim Biophys Acta.

[9] Xie Z, Zhang ZL, Hanzlik S, Cook E, Shen QXJ (2007). Salicylic acid inhibits gibberellin-induced alpha-amylase expression and seed germination via a pathway involving an abscisic-acid-inducible WRKY gene. Plant Mol Biol.

[10] Balazadeh S, Riano-Pachon DM, Mueller-Roeber B (2008). Transcription factors regulating leaf senescence in arabidopsis thaliana. Plant Biol.

[11] Guo Y, Cai Z, Gan S (2004). Transcriptome of Arabidopsis leaf senescence. Plant Cell Environ.

[12] Robatzek S, Somssich IE (2001). A new member of the Arabidopsis WRKY transcription factor family, AtWRKY6, is associated with both senescence- and defence-related processes. Plant J.

[13] Chen L, Xiang S, Chen Y, Li D, Yu D. Arabidopsis WRKY45 interacts with the DELLA protein RGL1 to positively regulate age-triggered leaf senescence. Mol Plant. 2017;10:1174-89. 10.1016/j.molp.2017.07.008 28735023

[14] Han M, Kim CY, Lee J, Lee SK, Jeon JS (2014). OsWRKY42 represses OsMT1d and induces reactive oxygen species and leaf senescence in rice. Mol Cells.

[15] Jing S, Zhou X, Song Y, Yu D (2009). Heterologous expression of OsWRKY23 gene enhances pathogen defense and dark-induced leaf senescence in Arabidopsis. Plant Growth Regul.

[16] Zhang H, Zhao M, Song Q, Zhao L, Wang G, Zhou C (2016). Identification and function analyses of senescence-associated WRKYs in wheat. Biochem Biophys Res Commun.

[17] Guillaumie S, Mzid R, Mechin V, Leon C, Hichri I, Destrac-Irvine A, Trossat-Magnin C, Delrot S, Lauvergeat V (2010). The grapevine transcription factor WRKY2 influences the lignin pathway and xylem development in tobacco. Plant Mol Biol.

[18] Li W, Tian ZX, Yu DQ (2015). WRKY13 acts in stem development in Arabidopsis thaliana. Plant Sci.

[19] Guo DS, Zhang JZ, Wang XL, Han X, Wei BY, Wang JQ, Li BX, Yu H, Huang QP, Gu HY (2015). The WRKY transcription factor WRKY71/EXB1 controls shoot branching by transcriptionally regulating RAX genes in Arabidopsis. Plant Cell.

[20] Guan Y, Meng X, Khanna R, LaMontagne E, Liu Y, Zhang S (2014). Phosphorylation of a WRKY transcription factor by MAPKs is required for pollen development and function in Arabidopsis. PLoS Genet.

[21] Wang X, Yan Y, Li Y, Chu X, Wu C, Guo X (2014). GhWRKY40, a multiple stress-responsive cotton WRKY gene, plays an important role in the wounding response and enhances susceptibility to ralstonia solanacearum infection in transgenic Nicotiana benthamiana. PLoS One.

[22] Jia H, Wang C, Wang F, Liu S, Li G, Guo X (2015). GhWRKY68 reduces resistance to salt and drought in transgenic Nicotiana benthamiana. PLoS One.

[23] Dou L, Zhang X, Pang C, Song M, Wei H, Fan S, Yu S (2014). Genome-wide analysis of the WRKY gene family in cotton. Molecular genetics and genomics: MGG.

[24] Gu L, Li L, Wei H, Wang H, Su J, Guo Y, Yu S (2018). Identification of the group IIa WRKY subfamily and the functional analysis of GhWRKY17 in upland cotton (Gossypium hirsutum L.). PLoS One.

[25] Lin M, Pang CY, Fan SL, Song MZ, Wei HL, Yu SX. Global analysis of the Gossypium hirsutum L. Transcriptome during leaf senescence by RNA-Seq. BMC Plant Biol. 2015;15:43. 10.1186/s12870-015-0433-5 PMC4342795 25849479

[26] Guo YF, Gan SS (2006). AtNAP, a NAC family transcription factor, has an important role in leaf senescence. Plant J.

[27] Noh YS, Amasino RM (1999). Identification of a promoter region responsible for the senescence-specific expression of SAG12. Plant Mol Biol.

[28] Chen Y, Wang Y, Huang J, Zheng C, Cai C, Wang Q, Wu CA (2017). salt and methyl jasmonate aggravate growth inhibition and senescence in Arabidopsis seedlings via the JA signaling pathway. Plant Sci.

[29] Robatzek S, Somssich IE (2002). Targets of AtWRKY6 regulation during plant senescence and pathogen defense. Genes Dev.

[30] Qiu K, Li ZP, Yang Z, Chen JY, Wu SX, Zhu XY, et al. EIN3 and ORE1 accelerate degreening during ethylene-mediated leaf senescence by directly activating chlorophyll catabolic genes in arabidopsis. PLoS Genet. 2015;11:e1005399. 10.1371/journal.pgen.1005399 PMC4517869 26218222

[31] Gao S, Gao J, Zhu X, Song Y, Li Z, Ren G, Zhou X, Kuai B (2016). ABF2, ABF3, and ABF4 promote ABA-mediated chlorophyll degradation and leaf senescence by transcriptional activation of chlorophyll catabolic genes and senescence-associated genes in Arabidopsis. Mol Plant.

[32] Robert N, Merlot S, N'Guyen V, Boisson-Dernier A, Schroeder JI (2006). A hypermorphic mutation in the protein phosphatase 2C HAB1 strongly affects ABA signaling in Arabidopsis. FEBS Lett.

[33] Bakshi M, Oelmüller R (2014). WRKY transcription factors. Plant Signal Behav.

[34] Sun J, An H, Shi W, Guo X, Li H (2012). Molecular cloning and characterization of GhWRKY11, a gene implicated in pathogen responses from cotton. S Afr J Bot.

[35] Karim A, Jiang Y, Guo L, Ling Z, Ye S, Duan Y, Li C, Luo K (2015). Isolation and characterization of a subgroup IIa WRKY transcription factor PtrWRKY40 from Populus trichocarpa. Tree Physiol.

[36] Shi WN, Liu DD, Hao LL, Wu CA, Guo XQ, Li H (2014). GhWRKY39, a member of the WRKY transcription factor family in cotton, has a positive role in disease resistance and salt stress tolerance. Plant Cell Tiss Org.

[37] Yan HR, Jia HH, Chen XB, Hao LL, An HL, Guo XQ (2014). The cotton WRKY transcription factor GhWRKY17 functions in drought and salt stress in transgenic Nicotiana benthamiana through ABA signaling and the modulation of reactive oxygen species production. Plant Cell Physiol.

[38] Matsui A, Ishida J, Morosawa T, Mochizuki Y, Kaminuma E, Endo TA, Okamoto M, Nambara E, Nakajima M, Kawashima M (2008). Arabidopsis transcriptome analysis under drought, cold, high-salinity and ABA treatment conditions using a tiling array. Plant Cell Physiol.

[39] Matzke AJ, Stoger EM, Schernthaner JP, Matzke MA (1990). Deletion analysis of a zein gene promoter in transgenic tobacco plants. Plant Mol Biol.

[40] Eulgem T, Somssich IE (2007). Networks of WRKY transcription factors in defense signaling. Curr Opin Plant Biol.

[41] Liu L, Xu W, Hu XS, Liu HJ, Lin YJ. W-box and G-box elements play important roles in early senescence of rice flag leaf. Sci Rep. 2016;6:20881. 10.1038/srep20881 PMC4749992 26864250

[42] Quirino BF, Noh YS, Himelblau E, Amasino RM (2000). Molecular aspects of leaf senescence. Trends Plant Sci.

[43] Thompson JE, Froese CD, Madey E, Smith MD, Hong YW (1998). Lipid metabolism during plant senescence. Prog Lipid Res.

[44] Lim PO, Kim HJ, Nam HG (2007). Leaf senescence. Annu Rev Plant Biol.

[45] Fischer AM (2012). The complex regulation of senescence. Crit Rev Plant Sci.

[46] Distelfeld A, Avni R, Fischer AM (2014). Senescence, nutrient remobilization, and yield in wheat and barley. J Exp Bot.

[47] Zhao FL, Ma JH, Li LB, Fan SL, Guo YN, Song MZ, Wei HL, Pang CY, Yu SX (2016). GhNAC12, a neutral candidate gene, leads to early aging in cotton (Gossypium hirsutum L). Gene.

[48] Fan K, Bibi N, Gan SS, Li F, Yuan SN, Ni M, Wang M, Shen H, Wang XD (2015). A novel NAP member GhNAP is involved in leaf senescence in Gossypium hirsutum. J Exp Bot.

[49] Palma-Guerrero J, Ma X, Torriani SFF, Zala M, Francisco CS, Hartmann FE, Croll D, McDonald BA (2017). Comparative transcriptome analyses in Zymoseptoria tritici reveal significant differences in gene expression among strains during plant infection. Mol Plant Microbe In.

[50] Wei X, Xu J, Guo H, Jiang L, Chen S, Yu C, Zhou Z, Hu P, Zhai H, Wan J (2010). DTH8 suppresses flowering in rice, influencing plant height and yield potential simultaneously. Plant Physiol.

[51] Xiang JS, Tang S, Zhi H, Jia GQ, Wang HJ, Diao XM. Loose Panicle1 encoding a novel WRKY transcription factor, regulates panicle development, stem elongation, and seed size in foxtail millet [Setaria italica (L.) P. Beauv.]. Plos One. 2017;12:e0178730. 10.1371/journal.pone.0178730 PMC5453597 28570666

[52] Zhang CQ, Xu Y, Lu Y, Yu HX, Gu MH, Liu QQ (2011). The WRKY transcription factor OsWRKY78 regulates stem elongation and seed development in rice. Planta.

[53] Navabi A, Iqbal M, Strenzke K, Spaner D (2006). The relationship between lodging and plant height in a diverse wheat population. Can J Plant Sci.

[54] Schmittgen TD, Livak KJ (2008). Analyzing real-time PCR data by the comparative C(T) method. Nat Protoc.

[55] Bent A (2006). Arabidopsis thaliana floral dip transformation method. Methods Mol Biol.

[56] Sparkes IA, Runions J, Kearns A, Hawes C (2006). Rapid, transient expression of fluorescent fusion proteins in tobacco plants and generation of stably transformed plants. Nat Protoc.

[57] Gao X, Britt RC, Jr., Shan L, He P. Agrobacterium-mediated virus-induced gene silencing assay in cotton. J Vis Exp. 2011;2938. 10.3791/2938 PMC3217629 21876527

[58] Gu Z, Huang C, Li F, Zhou X (2014). A versatile system for functional analysis of genes and microRNAs in cotton. Plant Biotechnol J.

[59] Shah ST, Pang CY, Fan SL, Song MZ, Arain S, Yu SX (2013). Isolation and expression profiling of GhNAC transcription factor genes in cotton (Gossypium hirsutum L.) during leaf senescence and in response to stresses. Gene.

