# Supplementary material for: Characterization and functional analysis of GhWRKY42, a group IId WRKY gene, in upland cotton (Gossypium hirsutum L.)
Source: BMC Genet. 2018 Jul 30;19:48. doi: 10.1186/s12863-018-0653-4 (PMC6065155; doi:10.1186/s12863-018-0653-4)
Supplement: Supplementary file 1 — Table S1. Predicted cis-acting elements in the promoter region of GhWRKY42. (DOCX 36 kb) [file 12863_2018_653_MOESM1_ESM.docx]

**Table S1. Predicted *cis*-acting elements in the promoter region of *GhWRKY42***

| **Cis-element** | **Position** | **Sequence (5'-3')** | **Function** |
| --- | --- | --- | --- |
| ***Stress responsive elements*** | | | |
| ABRE | -858(-) | TACGTG | Cis-acting element involved in the abscisic acid responsiveness |
| [AuxRR-core](http://bioinformatics.psb.ugent.be/webtools/plantcare/cgi-bin/show_site_info.htpl?QWhere=ID_of_Site%20like%20%27NT~AuxRR-core%27&StartAt=0&NbRecs=10) | -61(-) | GGTCCAT | Cis-acting regulatory element involved in auxin responsiveness |
| Box-W1 | -10(+),-1295(+) | TTGACC | Fungal elicitor responsive element |
| CGTCA-motif | -657(+) | CGTCA | Cis-acting regulatory element involved in the MeJA- responsiveness |
| ERE | -531(-) | ATTTCAAA | Ethylene-responsive element |
| GARE-motif | -461(+) | AAACAGA | Gibberellin-responsive element |
| HSE | -222(-),-950(+),-847(+),-502(+),-848(+) | AAAAAATTTC | Cis-acting element involved in heat stress responsiveness |
| MBS | -100(-) | TAACTG | MYB binding site involved in drought-inducibility |
| TCA-element | -266(-),-393(-) | GAGAAGAATA | Cis-acting element involved in salicylic acid responsiveness |
| TGA-element | -444(-) | AACGAC | Auxin-responsive element |
| TGACG-motif | -657(-) | TGACG | Cis-acting regulatory element involved in the MeJA-responsiveness |
| ***Light responsive elements*** | | | |
| 3-AF1 binding site | -1363(-) | TAAGAGAGGAA | Light responsive element |
| [4cl-CMA2b](http://bioinformatics.psb.ugent.be/webtools/plantcare/cgi-bin/show_site_info.htpl?QWhere=ID_of_Site%20like%20%27PC~4cl-CMA2b%27&StartAt=0&NbRecs=10) | -425(+) | TCTCACCAACC | Light responsive element |
| AT1-motif | -904(+) | ATTAATTTTACA | Part of a light responsive module |
| Box 4 | -561(+),-1009(-),-904(-),-1109(-) | ATTAAT | Part of a conserved DNA module involved in light responsiveness |
| Box I | -355(-),-531(-) | TTTCAAA | Light responsive element |
| CATT-motif | -802(-) | GCATTC | Part of a light responsive element |
| G-box | -858(+) | CACGTA | Cis-acting regulatory element involved in light responsiveness |
| GA-motif | -1369(-) | AAGGAAGA | Part of a light responsive element |
| GT1-motif | -103(-),-672(+),-372(+) | GGTTAA | Light responsive element |
| L-box | -425(+) | TCTCACCAACC | Part of a light responsive element |
| LAMP-element | -1319(+) | CTTTATCA | Part of a light responsive element |
| MRE | -369(-),-669(-) | AACCTAA | MYB binding site involved in light responsiveness |
| Sp1 | -1380(+) | CC(G/A)CCC | Light responsive element |
| TCT-motif | -425(+) | TCTTAC | Part of a light responsive element |
| ***Metabolism and development-related elements*** | | | |
| [AACA_motif](http://bioinformatics.psb.ugent.be/webtools/plantcare/cgi-bin/show_site_info.htpl?QWhere=ID_of_Site%20like%20%27OS~AACA_motif%27&StartAt=0&NbRecs=10) | -450(-) | TAACAAACTCCA | Involved in endosperm-specific negative expression |
| O2-site | -14(-) | GATGATGTGG | Cis-acting regulatory element involved in zein metabolism regulation |
| ***Others*** | | | |
| CCAAT-box | -434(-) | CAACGG | MYBHv1 binding site |
| AT-rich element | -980(-) | ATAGAAATCAA | Binding site of AT-rich DNA binding protein (ATBP-1) |
| MBS | -11(-) | CGGTCA | MYB Binding Site |
| W-box | -10(+)，-1295(+) | TTGACC |  |
