# Supplementary material for: Characterization and functional analysis of GhWRKY42, a group IId WRKY gene, in upland cotton (Gossypium hirsutum L.)
Source: BMC Genet. 2018 Jul 30;19:48. doi: 10.1186/s12863-018-0653-4 (PMC6065155; doi:10.1186/s12863-018-0653-4)
Supplement: Supplementary file 2 — Table S2. Primers used in this study. (DOCX 32 kb) [file 12863_2018_653_MOESM2_ESM.docx]

**Table S2. Primers used in this study**

| **Primer name** | **Sequence ( 5’- 3’)** | **Description** |
| --- | --- | --- |
| *GhActin*-F | ATCCTCCGTCTTGACCTTG | Primers used in qRT-PCR |
| *GhActin*-R | TGTCCGTCAGGCAACTCAT |  |
| *AtActin2-F* | AAGCTCTCCTTTGTTGCTGTT |  |
| *AtActin2-R* | GACTTCTGGGCATCTGAATCT |  |
| *GhWRKY42-F* | TTTTATGACGGTGAAAACAGGGG |  |
| *GhWRKY42-R* | ACGATGAACATGGCTGCTTACTATC |  |
| *GhNAP*-F | GCCCCAATTCACATGACACAGT |  |
| *GhNAP*-R | TCTCAACATGGTCACCTGTGGT |  |
| 42-F | ATTATATGGTTGGAGTTGGAT | T-vector construction |
| 42-R | CTATGACCATGATTACGCCAA |  |
| pBI121-42-XbaI-F | CTAGTCTAGAATGGCCGTTGAACTCATG | Overexpression vector construction |
| pBI121-42-SacI-R | CGAGCTCTTACGAAGATTCCAGGATGAGA |  |
| pBFP-42-XbaI-F | CTAGTCTAGAATGGCCGTTGAACTCATG | Subcellular localization |
| pBFP-42-SpeI-R | CTAGACTAGTCGAAGATTCCAGGATGAGA |  |
| pGBKT7-42-EcoRI-F | CCGGAATTCATGGCCGTTGAACTCATG | Transcriptional activity |
| pGBKT7-42-BamHI-R | CGCGGATCCTTACGAAGATTCCAGGATGAGA |  |
| VA-42-SpeI-F | CTAGACTAGTAAATGGAAGAAAACGCCGTCA | VIGS |
| VA-42-AscI-R | TCCCCCGGGTCGGTGTTGCGTAATAAACCT |  |
| Pro42-HindⅢ-F | TGGTCCACAAATAGAAATAGAGTT | Full-length promoter and deletion fragment construction |
| Pro42-1-HindⅢ-F | AGACATAGTTTTGACCGCATC |  |
| Pro42-2-HindⅢ-F | CCGTCTAATTTACTTTTTGAGAAGA |  |
| Pro42-3-HindⅢ-F | GAACTTATACATCGTAAAAATTAGAA |  |
| Pro42-XbaI-R-all | GTCTGCAACAACACCATTCA |  |
| 35S | GACGCACAATCCCACTATCC | Universal primers for the detection of positive clones |
| T7 | TAATACGACTCACTATAGGG |  |
